# Unveiling *Dirofilaria Asiatica* infection: first clinical insights and treatment challenges for this feline zoonotic parasitosis

**DOI:** 10.1007/s11259-025-10916-4

**Published:** 2025-09-30

**Authors:** Angel Almendros, Stefan Hobi, Zhou You, Tiffany Wong, Rebecca Traub

**Affiliations:** 1 Department of Veterinary Clinical Sciences, Jockey Club College of Veterinary Medicine and Life Sciences, City University of Hong Kong, Hong Kong SAR, China; 2 CityU Veterinary Medical Centre, City University of Hong Kong, Hong Kong SAR, China; 3 Department of Infectious Diseases and Public Health, Jockey Club College of Veterinary Medicine and Life Sciences, City University of Hong Kong, Hong Kong SAR, China

**Keywords:** Dirofilaria, Asiatica, Hongkongensis, Feline dirofilariasis, Zoonosis, Moxidectin, Subcutaneous nodules, Anaphylaxis, Respiratory distress

## Abstract

**Background:**

*Dirofilaria asiatica* (previously referred to as *Dirofilaria* sp. Hong Kong genotype, *Candidatus* Dirofilaria hongkongensis, or *Dirofilaria* sp. “hongkongensis”) is an emerging zoonotic filarioid nematode, initially described in human subcutaneous nodules in Hong Kong and later demonstrated in dogs and cats. This report includes the first description of clinical signs, diagnostic findings, including comparative clinicopathology, treatment and associated clinical complications in a feline infection.

**Methods:**

An 18-month-old, indoor-only spayed female Domestic Shorthair cat presented with subcutaneous nodules and microfilariae in blood smears. Diagnostic work-up included a PCR from blood, a quantitative modified Knott test, haematology, echocardiography, and abdominal ultrasonography.

**Results:**

PCR confirmed the presence of *Dirofilaria asiatica*. Haematology revealed neutrophilia (11.35 × 10³/µL) with left shift, and hyperproteinaemia (80 g/L). A quantitative modified Knott test revealed a microfilaremia of 36,907 per ml. Treatment with oral doxycycline and transdermal moxidectin triggered an acute onset of respiratory distress, managed with oxygen and dexamethasone. Nodules regressed, and microfilariae cleared by day 70.

**Conclusions:**

This case supports that indoor feline pets are susceptible to infection with *Dirofilaria asiatica*. In addition, we describe for the first time, the clinical and clinicopathological findings associated with infection and highlight the risks of anaphylactic reactions to microfilaricidal therapy. Moxidectin and doxycycline were critical for resolution, but pre-treatment with corticosteroids is recommended. Exorbitant microfilaremia might be a critical feature in cats. The zoonotic potential of this parasite warrants heightened surveillance in endemic regions.

## Background

*Dirofilaria* sp. Hong Kong genotype, recently classified as *Dirofilaria asiatica* (Colella et al. 2025), is a species that was first identified in 2012 from human subcutaneous nodules and subsequently detected retrospectively in dogs by PCR from histopathology samples, suggesting a zoonotic cycle involving vectors from the Culicinae and Anophelinae subfamilies (Capelli et al. 2018). Phylogenetically, it clusters within the *Nochtiella* subgenus, closely related to *D. repens*, but exhibits distinct genetic markers such as cox1 and 12 S rRNA sequences (Yilmaz et al. 2016; Pradeep et al. 2019). While *D. repens* primarily causes infections in dogs and humans across Europe and Asia, *Dirofilaria asiatica* appears restricted to Asia, with reports from India, Sri-Lanka, Bhutan (Gowrishankar et al. 2019; Atapattu et al. 2023; Huggins et al. 2024), Thailand, and more recently Hong Kong (Yilmaz et al. 2019; Manathunga et al. 2024), primarily affecting dogs and to a lesser degree cats. Zoonotic cases of *Dirofilaria asiatica* in humans have been reported in India and Thailand, and in returned travellers from Europe and Australia (Kumar et al. 2021; Winkler et al. 2017; Tirakunwichcha et al. 2022; Schroeder et al. 2023; Cope et al. 2024).

Recent studies confirmed *Dirofilaria asiatica* within subcutaneous nodules in one dog and a cat in Hong Kong (Manathunga et al. 2024). Nodules were located in the posterior part of the body, filaroids were identified by histopathological assessment and species confirmed by PCR. The nematodes had longitudinal cuticular ridges typical of the subgenus *Notchiella*, and the lesions resembled *D. repens*, consisting of granulomatous to pyogranulomatous dermatitis with eosinophilic infiltrates (Manathunga et al. 2024). Nevertheless, data regarding how *Dirofilaria asiatica* affects dogs or cats remains, however, scarce. Details on physical exams, clinical pathology, imaging studies and how dogs and cats respond to treatment has never been documented.

Diagnosis relies on PCR targeting cox1 and 12 S rRNA due to morphological overlap of microfilariae with those of *D. repens* (Yilmaz et al. 2016). Treatment has never been described in dogs and cats but might present challenges mirroring those of *D. immitis* in dogs, where potent microfilaricidal drugs such as diethylcarbamazine (DEC) can provoke fatal anaphylactic reactions owing to a sudden and massive destruction of circulating microfilariae (Atwell and Boreham 1983).

This case report offers novel insights into feline *Dirofilaria asiatica* infection, highlighting the challenges associated with its therapeutic management.

## Case presentation

**Fig. 1:**
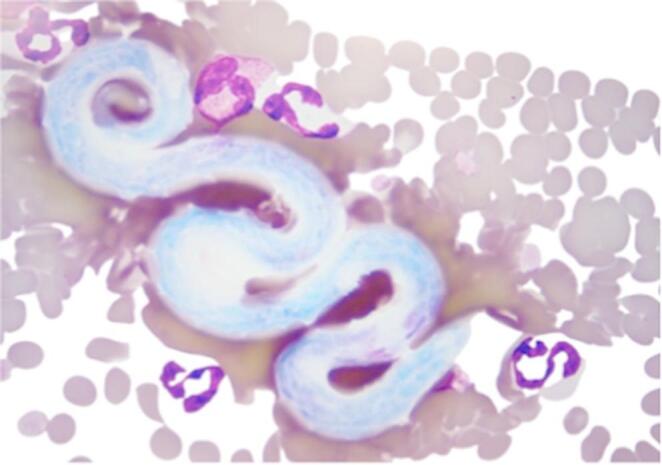
Stained blood smear (Diff-Quick). Microfilaria surrounded by red blood cells, neutrophils and an eosinophil from a cat blood sample (100x magnification)

An 18-month-old indoor-only spayed female Domestic Shorthair cat was referred to the Veterinary Medical Centre of City University of Hong Kong (VMC CityUHK) for further investigation of a 2-week history of subcutaneous nodules previously explored at the referring veterinarian, revealing the presence of a long, thin worm from one of the nodules at the left pinna. A blood smear had identified microfilariae but no further investigation had been performed. Other than intermittent fur ball vomiting, the cat was bright and alert, had a good appetite and no other gastrointestinal signs were reported. On day 1 (D1) the body condition score (BCS) was 5/9 with a weight of 4.14 kg. Initial physical and dermatological examination revealed two palpable subcutaneous, intact and non-moveable nodules, including a small, raised nodule (4 mm) on the left elbow region, and a larger nodule (9 mm) on the left pinnae. The nodules were reported not to be pruritic or painful. Cardiothoracic auscultation was unremarkable, with a heart rate (HR) of 186 bpm, and a respiratory rate (RR) of 32 bpm. Her mucous membranes were pink and moist, and no obvious pulse deficits were detected. The rest of the physical exam was unremarkable. An in-house blood smear exam revealed numerous microfilariae morphologically suggestive of filarial species (Fig. 1). Blood samples were submitted to the Veterinary Diagnostic Laboratory of City University of Hong Kong (VDL CityUHK) for a complete cell count using an automated machine (Siemens ADVIA 2120i Hematology System, Munich, Germany) and performing a differential cytology interpreted by a pathologist. Further sampling was submitted to the Department of Infectious Diseases and Public Health of CityUHK for a Knott test and PCR assay for identification of microfilariae.

**Fig. 2:**
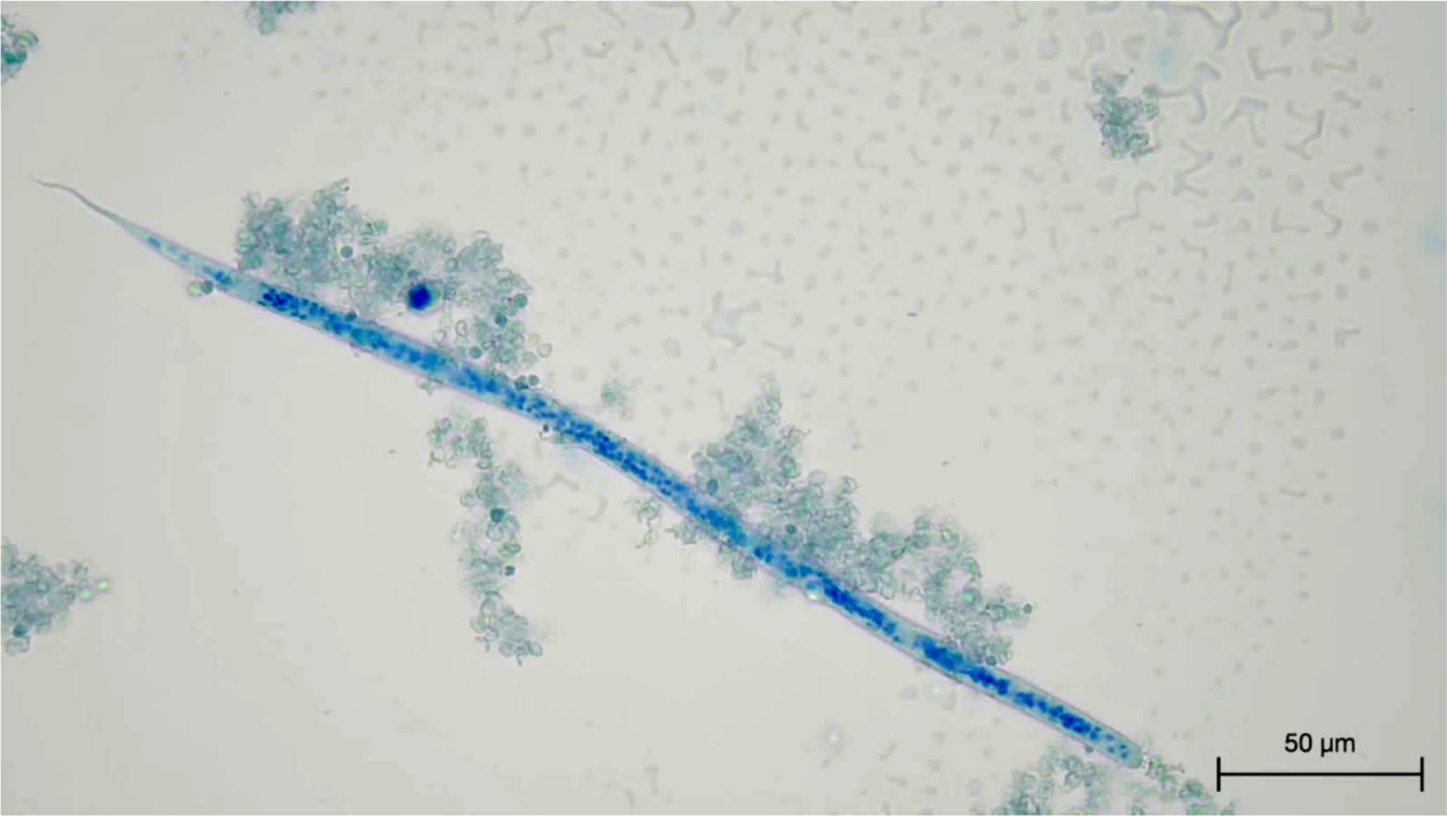
Modified Knott test. Microfilaria fixed in 2% formalin and stained with methylene blue displaying curved tail, blunt head and 3 cephalic nuclei from a cat blood sample (40x magnification)

On day 2, the results of the haematological assessment revealed neutrophilia (11.35 × 10³/µL; reference interval - RI, 2.30–10.29) with a left shift (bands: 0.15 × 10³/µL; RI, 0.00–0.10), hyperproteinaemia (80 g/L; RI, 59–75), and a normal eosinophil count (0.92 × 10³/µL; RI, 0.10–1.80). For quantitation and species identification, 1 ml of whole blood was subjected to a Modified Knott Test (MKT) stained with 0.1% methylene blue (Genchi et al. 2007). The average count across three 10 07*µl* aliquots of the MKT sediment revealed a microfilarial count of 36,907/ml of blood. Microfilariae were unsheathed, with a blunt head and curved tail (Fig. 2) as per previous descriptions of *Dirofilaria* sp. Hong Kong genotype *asiatica* sp. nov. (Atapattu et al. 2023).

For molecular characterization, DNA was extracted from whole blood using the DNeasy Blood and Tissue Kit (Qiagen, Germany) according to the manufacturer’s protocols.

Conventional PCR amplification was performed targeting a 763 bp region of the mitochondrial (mt) cytochrome oxidase – 1 (cox-1) gene using primers Diro-cox1-F (5′-GCT TTG TCT TTT TGG TTT ACTTTT − 3′) and Diro-cox1-R (5′-TCA AAC CTC CAA TAG.

TAA AAA GAA − 3′) (Pradeep et al. 2019). PCR reactions were carried out in a final volume of 20 µl, containing 10 µl 2X Phanta Flash Master Mix (Vazyme, China), 1 µl of each primer (10 µM), 6 µl distilled water and 2 µl of template DNA. The mixtures were amplified using 35 cycles under the following conditions: denaturation at 98 °C for 40 s, annealing at 58 °C for 30 s, and extension at 72 °C for 1 min, followed by a final extension at 72 °C for 1 min using the SimpliAmp thermocycler (Thermofisher, USA). The PCR products were visualized after electrophoresis using 2% agarose gel and submitted for bidirectional Sanger sequencing at BGI Genomics (Guangdong, China). Clear and readable sequences of the 763 bp region of the cox gene were directly compared with reference sequences using BLASTn, which revealed between 99.86 and 100% sequence identity with *Dirofilaria* sp. ‘*hongkongensis*’ over 88–96% sequence coverage, isolated from cats, dogs and humans originating in India and in Hong Kong (GenBank accession numbers PQ327004-5, NC031365, OL744441).

The PCR result was available on day 15 and the patient returned on day 17 for further evaluation. The cat was bright and alert, eating well, with a BCS of 5/9, and a body weight of 4.04 kg. A new nodule had developed on her right forelimb of approximately 5 mm, while the previously reported nodules on the left forelimb and the left pinnae remained unchanged. An echocardiograph was unremarkable with no visible adult parasites in the heart chambers or in the pulmonary artery as it might occur in cases of *D. immitis* infection. An abdominal ultrasound to rule out other comorbidities or potential signs of visceral migration of the parasite showed mild mid abdominal lymphadenopathy but there was no evidence of visceral parasitosis. Haematology and biochemistry were unremarkable and a urine analysis revealed a urine specific gravity of 1.016, protein traces and a pH of 6 (RI, 6–7).

**Fig. 3:**
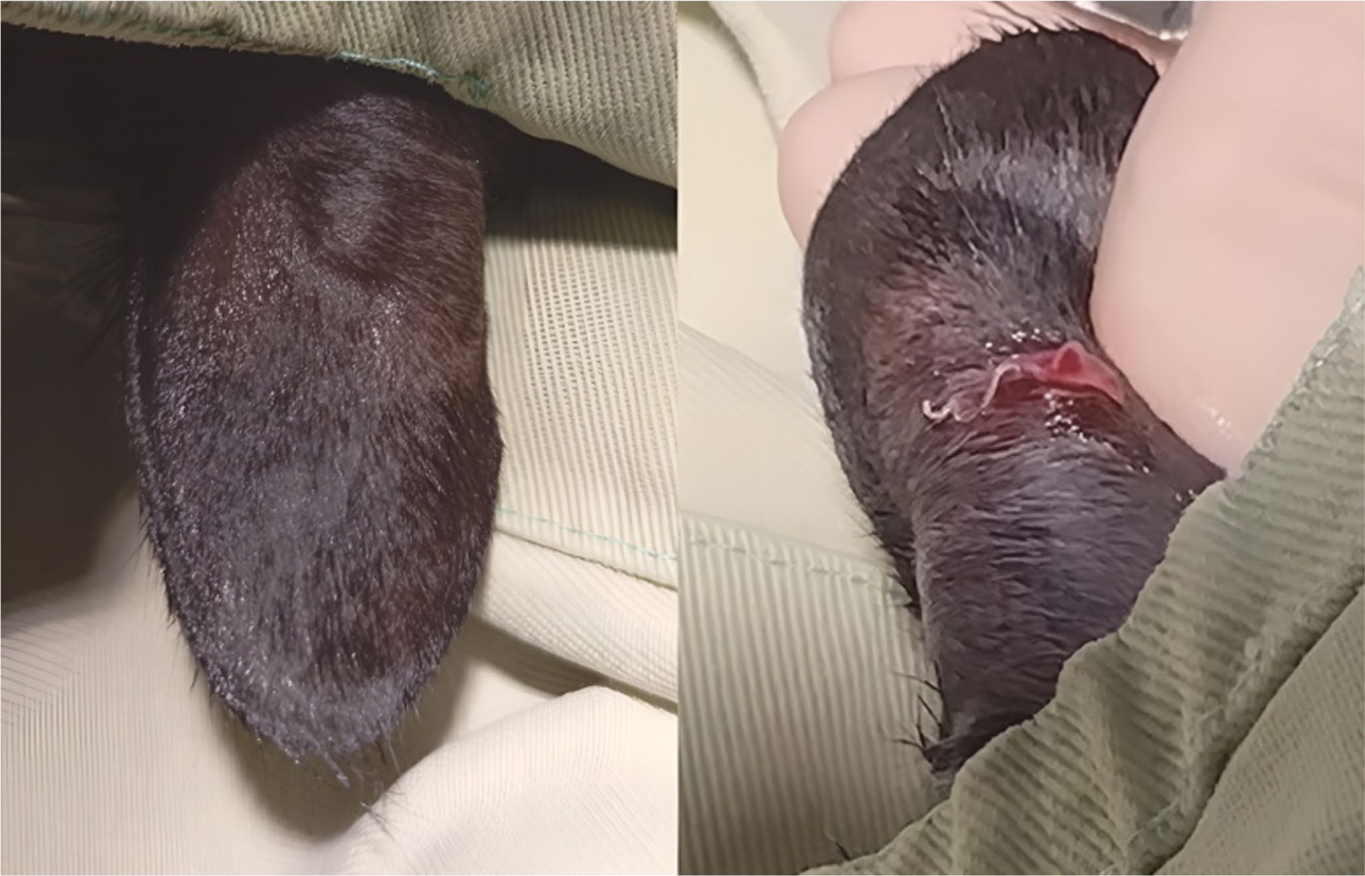
Exploration of a nodule on the left pinna of a cat containing and adult filaroid

For further exploration of the cutaneous nodules, the patient was sedated intramuscularly with butorphanol (Torbugesic, Zoetis, Australia) 0.3 mg/kg and dexmedetomidine (Dexdomitor, Zoetis, Australia) 6 µg/kg. One large live filaroid was extracted from the ear nodule and was submitted for pathological examination (Fig. 3). After confirming the absence of worms within the cardiovascular system, the patient was prescribed a transdermal microfilaricidal drug, used routinely in cats for endo and ectoparasite prevention containing imidacloprid 10% and moxidectin 1% (Advocate, Elanco, USA), administered off-label monthly, as a microfilaricide and an adulticide (Petry et al. 2015). Additionally, oral doxycycline (Vibramycin, Pfizer, USA) was prescribed for 30 days at 10 mg/kg q 24 h to target *D. asiatica*’s endosymbiont Wolbachia (Kamkong et al. 2023), which has been demonstrated to improve the microfilaricidal and adulticidal efficacies of the macrocyclic lactone (Bazzocchi et al. 2008; Savadelis et al. 2017).

**Fig. 4:**
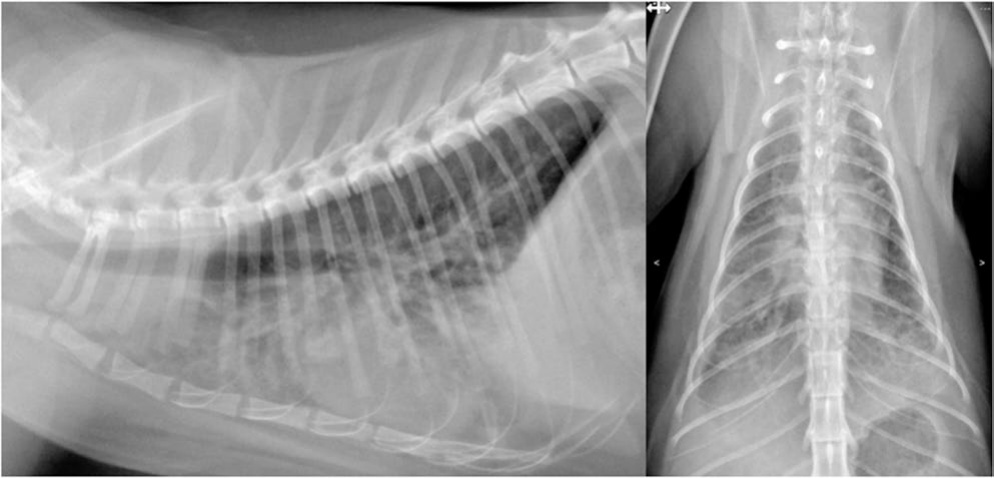
Radiographic interstitial lung pattern in a microfilaremic cat following moxidectin and doxycycline treatment. Right lateral view on the left and dorsoventral view on the right

The following day (D18), prednisolone (Macrolone, Mavlab, Australia) 2 mg/kg was additionally prescribed due to concerns regarding the effects of microfilarial death causing the increased respiratory rate reported by the owner, but the drugs were never collected. The cat presented to the emergency department on the evening of day 18, approximately 30 h post-treatment, after developing an onset of acute respiratory distress with a RR of 92 bpm, oxygen saturation (SpO₂) of 83%, and diffuse interstitial lung patterns on radiography (Fig. 4). The cat was admitted to the hospital and was initially managed with oxygen therapy (15 L/min), dexamethasone (Dexafort, Merck, USA) 0.1 mg/kg IV q12h, and ampicillin-sulbactam (Unasyn, Pfizer, USA) 30 mg/kg IV q8h.

Initial blood work upon admission revealed mild hyperglycaemia (11.34 mmol/L; RI, 4.11–8.84), likely due to stress, along with a mild decrease in urea (4.7 mmol/L; RI, 5.7–12.9). The white blood cell count was normal, but the patient was mildly neutropenic (2.11 × 10³/µL; RI, 2.30–10.29) and exhibited mild eosinopenia (0.08 × 10³/µL; RI, 0.17–1.57). Blood gas (venous) values included pH 7.31 (RI, 7.22–7.38), HCO3 19.5 mmol/L (RI, 15.4–23.4), pCO2 41.1 mmHg (RI, 32.8–50.8), pO2 33.3 mmHg (27.6–49.6). Oximetry parameters in venous blood included cHb 10.9 g/dL (9.8–15.4), sO2 43.4% (62.4 +/- 13.5), and the metabolite Lactate measurement was 2 mmol/L (de Morais and Dibartola 2017; Tamura et al. 2015).

During hospitalization, a nasogastric tube (NGT) was inserted for nutritional support. Supportive treatment included 8 ml/hr IV Ringer’s lactate infusion solution (LRS), 0.1 mg/kg dexamethasone (Dexafort, Merck, USA) q12h IV, 30 mg/kg ampicillin-sulbactam (Unasyn, Pfizer, USA) q8h IV, 10 mg/kg doxycycline (Vibramycin, Pfizer, USA) q24h via NGT, 0.02 mg/kg buprenorphine (Zorbium, Elanco, USA) q6h IV, and 1 mg/kg maropitant (Cerenia, Zoetis, USA) q24h IV. The oxygen level was adjusted hourly, ranging from 10 to 15 L/min.

The cat was discharged 7 days after admission when the RR returned to normal.

On day 41, the patient returned for a blood test. Samples were collected in EDTA for a blood smear examination and a test for the detection of microfilariae. A microscopical assessment in-house revealed absence of microfilariae under stained and fresh blood smear exams. The sample was submitted for further analysis to a referral laboratory, and resolution of infection was confirmed by a clear MKT and a negative PCR on day 65. Nodules regressed by day 70. A final check-up on day 72 revealed no abnormalities on echocardiogram or abdominal ultrasound. The patient was discharged from the clinic with instructions to continue long-term application of imidacloprid 10% and moxidectin 1% spot-on formulation (Advocate, Elanco, USA). No recurrence of clinical signs was observed over 3 months after the last check-up.

## Discussion

This case unveils for the first time a comprehensive clinical description of *Dirofilaria asiatica* on a feline patient (*Felis catus*) together with treatment outcome. Other than the description of histopathological lesions and molecular confirmation, little has been reported about this parasite and its effect on dogs and cats (To et al. 2012; Manathunga et al. 2024). Weight loss and vomiting were mentioned in the history notes in one cat in a previous study, but no other diagnostic data was available to correlate the clinical signs with the parasite infection (Manathunga et al. 2024). The present case additionally highlights potential life-threatening complications after the use of a macrocyclic lactone, a common treatment for ectoparasites, gastrointestinal nematodes and prophylaxis for heartworm (Imidacloprid 10% and moxidectin 1% spot-on formulation), that was used for its known microfilaricidal and long-term adulticidal effects.

This case supports previous findings where felines expanded the known host range beyond humans and dogs for *Dirofilaria asiatica* (Manathunga et al. 2024; To et al. 2012). A clinicopathological overlap was demonstrated by the cat’s subcutaneous nodules and microfilariae, mirroring *D. repens* infections and further emphasizing the need for molecular differentiation in endemic regions (Ferri et al. 2009). In the present case, PCR unequivocally identified *Dirofilaria asiatica.* Unlike *D. immitis*, *Dirofilaria asiatica* exhibits tropism for subcutaneous tissues but absence of cardiac involvement, corroborating prior reports (To et al. 2012; Manathunga et al. 2024).

Serum chemistry was unremarkable in this case, with marginal hyperglycaemia and hyperproteinaemia likely due to stress and an ongoing inflammatory process. Neutrophilia and a left shift were present and were interpreted as an active inflammatory response. Other than occasional furball-associated vomiting, no clinical signs were associated with this cutaneous filarioid infection. The presence of mild mid-abdominal lymphadenopathy was suspected to be reactive and considered a non-specific finding. However, previous reports of *D. repens* and *Dirofilaria asiatica* have also identified lymphadenopathy (Manathunga et al. 2024; To et al. 2012; Pampiglione and Rivasi 2000). Reactive lymphadenopathy is not uncommon and could be expected in young cats due to antigenic stimulation with or without gastrointestinal signs such as intermittent vomiting (Perlini et al. 2018; Griffin 2021). Feline immunodeficiency virus (FIV) and feline leukaemia virus (FeLV) tests were negative in our cat, which did not show any obvious co-morbidities or co-infections as it might be seen in other cases of *Dirofilaria* infection (Carbonara et al. 2025).

The location of the skin lesions in this case were different to a previous study, where all cases including 4 male cats and a female dog presented lesions in the posterior part of the body (Manathunga et al. 2024). Different to previous reports the cat affected here was a female. In this case the skin lesions were in the anterior part of the body, at the left pinna and both elbow regions, which would align with the biting preferences of mosquitos. Interestingly, in the current case the nodules were neither pruritic nor painful, which is in contrast to other cases of cutaneous dirofilariasis where pruritus is commonly seen and may even mimic allergic dermatitis (Tarello 2011). Whether this is associated with a reduced movement of *Dirofilaria asiatica*, a modified expression of secretion products or individual factors, needs to be further elucidated.

Treatment complications might have occurred due to an immunologic reaction demonstrated by the severe respiratory distress following moxidectin and doxycycline application. This has been observed with *D. immitis* treatment in dogs linked to a sudden and severe destruction of circulating microfilariae, most frequently seen with the administration of DEC (Atwell and Boreham 1983). Corticosteroids were critical to mitigating this reaction. Although macrocyclic lactones are significantly safer than DEC in attaining a more gradual reduction in microfilarial clearance, the extremely high microfilarial count in this cat coupled with the use of doxycycline in combination with moxidectin, could have potentiated this reaction. Macrocyclic lactones in combination with doxycycline are known to have significant higher microfilaricidal effects compared to macrocyclic lactones alone (Kramer et al. 2018; Bazzocchi et al. 2008; Savadelis et al. 2017). This warrant caution due to the risk of anaphylaxis. Treating with macrocytic lactones alone and pretreatment with prednisolone or dexamethasone is advisable in future cases.

This case alerts clinicians for potential acute respiratory crises following the treatment with preventative care drugs such as moxidectin that can be further exacerbated with the administration of concurrent doxycycline. Since antigen tests are not available for this nematode species, screening for microfilarial identification by microscopy might be a sensible option in cats, especially if they have skin nodules or are exposed to mosquitos. Microfilariae presence would prompt the use of molecular testing for confirmation.

There are several limitations of this study. Being a case report, this study was retrospective in nature. Additionally, this case report included only one case, lacking any control and leading to potential interpretation bias that might not be representative for a general population of cats infected with the same nematode. Another limitation was the lack of skin biopsies due to owner refusal. A nematode could be directly isolated from associated lesions, and a granulomatous inflammation, reported in previous studies, was suspected based on the appearance of lesions but could not be confirmed histopathologically.

The blood gas analysis was from venous origin in this case, hence the ranges are adjusted. This explains the oxygenation discrepancy in blood gas PO2 versus sPO2, which is only reliable in arterial samples. However, ventilation and acid-base can be estimated and evaluated respectively in venous samples when arterial samples are not available (de Morais and Dibartola 2017).

In this case we emphasize the zoonotic and epidemiological significance of felines as potential reservoirs for *Dirofilaria asiatica*. Given the potential high microfilarial counts, surveillance and monthly prophylaxis for Hong Kong’s feline population are crucial to mitigate risks to the public. The summer onset of symptoms in this case report coincides with the peak of the mosquito activity. Vector dynamics therefore are important and suggest summer months to be a higher risk for transmission (To et al. 2012; Otranto et al. 2009).

## Conclusion

This case highlights *Dirofilaria asiatica* as an emerging feline pathogen with zoonotic potential. Affected individuals may only show subcutaneous nodules, mimicking neoplastic, inflammatory or other infectious pathologies, underlining the need for further diagnostics. Treatment requires multimodal therapy to address both parasitosis and immunologic complications. Public health messaging should emphasize on year-round parasite and vector prevention in pets, particularly in endemic regions like Hong Kong.

## Data Availability

Data are available upon request, with patient identifiers redacted.
